# Genomic Sequencing of sod1D Yeast That Escape Spore Death

**DOI:** 10.1093/geroni/igab046.3608

**Published:** 2021-12-17

**Authors:** Temiloluwa Adanlawo, Helen Vander Wende

**Affiliations:** 1 Howard University, Washington, District of Columbia, United States; 2 University of California Berkeley, Berkeley, California, United States

## Abstract

Amyotrophic lateral sclerosis (ALS) is a devastating neurodegenerative disease that impacts nerve cells and the spinal cord, which in some cases are linked to mutations in the Superoxide Dismutase 1 (SOD1) gene. Sod1 is an antioxidant within cells that converts reactive oxygen from superoxide into water using a copper and zinc ion to deactivate the oxygen. When the SOD1 gene is deleted, yeast cells are still able to undergo meiotic divisions and generate four spores, but the spores that are produced are inviable. However, we see that randomly, sod1∆ spores can grow on rich media. This leads us to hypothesize that somewhere in the genome, there is a suppressor mutation that allows these cells to grow. We tested this hypothesis by preparing samples for whole genome sequencing. By comparing the genomic sequences from our suppressor mutants to wild-type controls, we’re able to identify a single point mutation within a gene called NCA2, which codes for a protein that regulates expression of Fo-F1 ATP synthase subunits 6 and 8. Given this result, we are now working to try and understand the relationship between the sod1∆ spore death phenotype and the modulation of ATP synthase activity. In summary, the results from our work have the potential to further help us understand what role Sod1 plays in yeast meiosis and may be able to give us a deeper understanding for ALS cases that are linked to Sod1.

